# Heterogeneous clinical features in Cockayne syndrome patients and siblings carrying the same *CSA* mutations

**DOI:** 10.1186/s13023-022-02257-1

**Published:** 2022-03-05

**Authors:** Asma Chikhaoui, Ichraf Kraoua, Nadège Calmels, Sami Bouchoucha, Cathy Obringer, Khouloud Zayoud, Benjamin Montagne, Ridha M’rad, Sonia Abdelhak, Vincent Laugel, Miria Ricchetti, Ilhem Turki, Houda Yacoub-Youssef

**Affiliations:** 1 Laboratory of Biomedical Genomics and Oncogenetics (LR20IPT05), Institut Pasteur de Tunis, Université Tunis El Manar, El Manar I, BP 74, 13 Place Pasteur, 1002 Tunis-Belvedere, Tunisia; 2 LR18SP04 and Department of Child and Adolescent Neurology, National Institute Mongi Ben Hmida of Neurology, 1007 Tunis, Tunisia; 3 Laboratoires de Diagnostic Génétique, Institut de Génétique Médicale d’Alsace, Nouvel Hôpital Civil, Hôpitaux Universitaires de Strasbourg, Strasbourg, France; 4 Laboratoire de Génétique Médicale, INSERM UMR1112, Institut de Génétique Médicale d’Alsace, Faculté de Médecine de Strasbourg, Hôpitaux Universitaires de Strasbourg, Strasbourg, France; 5 Service Orthopédie, Hôpital d’enfant Béchir Hamza, Tunis, Tunisia; 6 Institut Pasteur, Team Stability of Nuclear and Mitochondrial DNA, Stem Cells and Development, UMR 3738 CNRS, 25-28 rue du Dr. Roux, 75015 Paris, France; 7 Service des Maladies Congénitales et Héréditaires de l’Hôpital Charles Nicolle, Tunis, Tunisia; 8 Human Genetics Laboratory (LR99ES10), Faculté de Médicine de Tunis, LR99ES10 Human Genetics Laboratory, Université Tunis El Manar, 1007 Tunis, Tunisia

**Keywords:** Cockayne syndrome, Targeted gene sequencing, *ERCC8*, CSA, Clinical heterogeneity, Siblings

## Abstract

**Background:**

Cockayne syndrome (CS) is a rare autosomal recessive disorder caused by mutations in *ERCC6/CSB* or *ERCC8/CSA* that participate in the transcription-coupled nucleotide excision repair (TC-NER) of UV-induced DNA damage. CS patients display a large heterogeneity of clinical symptoms and severities, the reason of which is not fully understood, and that cannot be anticipated in the diagnostic phase. In addition, little data is available for affected siblings, and this disease is largely undiagnosed in North Africa.

**Methods:**

We report here the clinical description as well as genetic and functional characterization of eight Tunisian CS patients, including siblings. These patients, who belonged to six unrelated families, underwent complete clinical examination and biochemical analyses. Sanger sequencing was performed for the recurrent mutation in five families, and targeted gene sequencing was done for one patient of the sixth family. We also performed Recovery RNA Synthesis (RRS) to confirm the functional impairment of DNA repair in patient-derived fibroblasts.

**Results:**

Six out of eight patients carried a homozygous indel mutation (c.598_600delinsAA) in exon 7 of *ERCC8*, and displayed a variable clinical spectrum including between siblings sharing the same mutation. The other two patients were siblings who carried a homozygous splice-site variant in *ERCC8* (c.843+1G>C). This last pair presented more severe clinical manifestations, which are rarely associated with *CSA* mutations, leading to gastrostomy and hepatic damage. Impaired TC-NER was confirmed by RRS in six tested patients.

**Conclusions:**

This study provides the first deep characterization of case series of CS patients carrying *CSA* mutations in North Africa. These mutations have been described only in this region and in the Middle-East. We also provide the largest characterization of multiple unrelated patients, as well as siblings, carrying the same mutation, providing a framework for dissecting elusive genotype–phenotype correlations in CS.

**Supplementary Information:**

The online version contains supplementary material available at 10.1186/s13023-022-02257-1.

## Background

Nucleotide Excision Repair (NER) is a complex DNA repair system capable of removing a variety of DNA lesions such as UV-induced photolesions and chemical adducts [1]. Deficiency in one of the proteins implicated in NER can result in heterogeneous rare disorders such as Xeroderma pigmentosum (XP), Trichothiodystrophy (TTD), and Cockayne syndrome (CS) [2]. Some of these diseases are frequently associated with neurodegeneration (TTD, CS), and others with cancer predisposition (XP, but not CS). CS patients display characteristics of accelerated aging, therefore this disease is defined as a segmental progeroid syndrome. CS is characterized by growth failure, progressive neurologic dysfunction, microcephaly, and intellectual disability along with other defects such as cutaneous photosensitivity, kyphosis, ankylosis, and optic atrophy [3]. The phenotype of CS patients varies widely, and phenotype-genotype correlations remain elusive [4]. The phenotypic variability of CS patients is challenging also for establishing a diagnosis and supportive treatments. Patients with the most severe forms exhibit a very short life expectancy (8.4 years) [5], and no cure is available to date.

In Europe, the incidence of this syndrome is estimated to less than 2.7 per million [6]. Although the incidence rates of other DNA repair disorders such XP have been reported in Tunisia [7], no epidemiological data are available in other North African countries, and they are missing for CS throughout the region.

To date, genetic investigations have identified two genes associated with CS: *ERCC8* (OMIM: 216400) that codes for the CSA protein, and *ERCC6* (OMIM: 133540) that codes for the CSB protein. Furthermore, an extremely rare combined form of XP/CS has been associated with *XPG* mutations [8]. The *ERCC6/CSB* gene is localized in the chromosomal region 10q11 and harbors 23 exons. In a recent study, 102 variations were reported, that mostly consisted of nonsense mutations and frameshifts [9]. A more recent study from our consortium has identified a novel *ERCC6/CSB* mutation in three unrelated Tunisian patients [10]. The *ERCC8/CSA* gene is localized in the chromosomal region 5q12.1, contains 13 exons, and 38 variations have been reported, which are also largely composed of missense mutations and deletions [9].

The CSB protein plays multiple roles: in addition to initiate TC-NER, it has an ATPase-dependent chromatin remodeling activity, is involved in another type of DNA repair (base excision repair, or BER), and is implicated in transcription regulation [11]. The CSA protein forms a complex with the DDB1-CUL4-based E3 ubiquitin ligase complex [12, 13] that is activated in response to UV irradiation, and is essential for the recruitment of the TC-NER protein machinery. Mutations in *ERCC8/CSA* generally result in the common CS type I, which is less severe than CS type II, with symptoms appearing in the first years of life [14].

In Tunisia and North Africa, genetic diagnosis of CS is not performed due to the absence of referral centers. A few studies reported fragmented and incomplete clinical aspects, which, however, were not sufficient to help clinicians identifying the disease and ensuring early diagnosis. Recently, we comprehensively described of a novel *ERCC6/CSB* mutation in Tunisian patients.

Here, we report an extensive clinical description and conduct genetic and functional investigations of eight CS patients mutated in *ERCC8/CSA* to accurately characterize the disease in the Tunisian population. This study represents the largest cohort reported in the region, and also describes two cases of siblings as well as multiple patients carrying the same mutation, which is relevant for global investigation of genotype/phenotype correlations in CS.

## Results

### Clinical features of CS-A patients

The clinical characterization of the eight CS patients is summarized in Table 1.

**Table 1:** Clinical, biological, imaging and genetic findings of patients with CS

Code family	Code patient	Country	Geographic origin	Sex	Age at first symptoms (months)	Age at diagnosis (years, months)	Consanguinity/Endogamy	Siblings	Clinical classification
CS1	EA1	Tunisia	North West	F	12	5	Endogamous	Yes	CSII
CS1	EA2	Tunisia	North West	M	12	1.6			CSII
CS2	EA	Tunisia	South	M	6	4	Consanguineous		CSI
CS6	EA1	Tunisia	North West	M	24	3	Consanguineous	Yes	CSI
CS6	EA2	Tunisia	North West	M	17	1.5			CSI
CS7	EA	Tunisia	North West	M	12	4	Endogamous		CSI
CS11	EA	Tunisia	North West	M	Birth	1.8	Consanguineous		CSII
CS16	EA	Tunisia	North West	F	5	7	Consanguineous		CSI

Code family	Mutation genomic DNA (homozygous)	Protein mutation	Gastrostomy	First symptoms	Prenatal abnormalities			
					IUGR	Microcephaly	Cerebellar hypoplasia	Oligoamnios
*CS1*	*c.843 + 1G > C*	p.Ala240Glyfs*8	*+*	*PMD, GD, microcephaly*	*+*	*−*	*−*	*−*
*CS1*	*c.843 + 1G > C*	p.Ala240Glyfs*9	*−*	*PMD, GD, microcephaly*	*+*	*+*	*−*	*+*
CS2	c.598_600delinsAA	p.Tyr200Lysfs*12	−	GD	−	−	−	−
*CS6*	*c.598_600delinsAA*	p.Tyr200Lysfs*12	*−*	*PMD*	*−*	*−*	*−*	*−*
*CS6*	*c.598_600delinsAA*	p.Tyr200Lysfs*12	*−*	*PMD*	*−*	*−*	*−*	*−*
CS7	c.598_600delinsAA	p.Tyr200Lysfs*12	−	PMD	−	−	−	−
CS11	c.598_600delinsAA	p.Tyr200Lysfs*12	−	Arthrogryposis	+	−	−	−
CS16	c.598_600delinsAA	p.Tyr200Lysfs*12	−	PMD	+	−	−	−

Code family	Code patient	Birth findings			Post-natal findings (years.months)			Dysmorphism					
		Birth weight (g)	Birth height (cm)	Head circumference at birth (cm)	Weight (kg)	Height (cm)	Head circumference (cm)	Enophtalmia	Thin skin	Bird like nose			
*CS1*	*EA1*	*2300*	*49*	*34*	*3y.4 m; 12 (− 3SD)*	*95 (normal)*	*43 (− 4SD)*	*+*	*+*	*+*			
*CS1*	*EA2*	*2550*	*45.5*	*NA*	*1y.3 m; 7*	*NA*	*40 (− 4SD)*	*+*	*+*	*−*			
CS2	EA	2450	48	32.5	3y.7 m; 10 (− 4SD)	68 (− 6SD)	43 (− 5SD)	+	+	+			
*CS6*	*EA1*	*3300*	*51*	*35*	*4y.6 m; 14 (− 2SD)*	*98 (normal)*	*47 (− 3SD)*	*+*	*+*	*−*			
*CS6*	*EA2*	*3400*	*50*	*34*	*2y; 11 (− 2SD)*	*83 (− 1SD)*	*46 (− 2SD)*	*+*	*+*	*−*			
CS7	EA	2650	49	34	4y; 10 (− 4SD)	89 (− 3SD)	45 (− 4SD)	+	+	+			
CS11	EA	2500	44	31	1y.8 m; 6.8 (− 4SD)	71 (normal)	41 (− 3SD)	+	+	+			
CS16	EA	1450	39	28	7y.5 m; 9 (− 3SD)	82 (− 3SD)	38 (− 2SD)	+	+	+			

Code family	Neurological findings												
	Microcephaly	Psychomotor delay	Independent Sitting (months)	Independent walking (years)	Mental retardation	Limb spasticity	Contractures	Pyramidal signs	Neurogenic signs	Ataxia	Extrapyramidal signs	Epilepsy	Behavioral abnormalities
*CS1*	*+*	*+*	*18*	*+ (2.5)*	*+*	*+*	*+*	*+*	*−*	*+*	*−*	*−*	*+ (irritability)*
*CS1*	*+*	*+*	*NA*	*NA*	*+*	*−*	*−*	*+*	*−*	*−*	*−*	*−*	*−*
CS2	+	+	18	−	+	+	+	+	−	−	−	−	−
*CS6*	*+*	*+*	*9*	*2*	*+*	*+*	*+*	*+*	*−*	*−*	*−*	*−*	*−*
*CS6*	*+*	*+*	*8*	*−*	*+*	*+*	*+*	*+*	*−*	*−*	*−*	*−*	*−*
CS7	+	+	20	3	+	+	+	+	−	+	−	−	−
CS11	+	+	−	−	+	+	+	+	+	+	−	−	−
CS16	+	+	−	−	+	+	+	+	−	−	−	−	−

Code family	Code patient	Ophthalmological findings			Otoralyngological findings		Dermatological findings					
		Cataracts	Optic atrophy	Pigmentary retinopathy	Sensorineural deafness	Auditory evoked response	Photosensitivity	Eczema	Thin skin	Pigmentation abnormalities	Hair abnormalities	Nail abnormalities
*CS1*	*EA1*	*−*	*−*	*−*	*+*	*90 dB*	*−*	*−*	*+*	*+*	*+*	*−*
*CS1*	*EA2*	*+*	*−*	*−*	*+*	*50/60 Db*	*−*	*−*	*+*	*+*	*−*	*−*
CS2	EA	−	−	+	+	50 dB	+	−	+	−	−	−
*CS6*	*EA1*	*−*	*−*	*−*	*+*	*90/100 dB*	*+*	*−*	*+*	*+*	*−*	*−*
*CS6*	*EA2*	*−*	*−*	*−*	*+*	*NA*	*+*	*−*	*+*	*+*	*−*	*−*
CS7	EA	+	−	−	+	70 dB	+	−	+	−	−	−
CS11	EA	+	−	−	− (18 months)	NA	−	−	+	−	−	−
CS16	EA	+	−	−	+	NA	+	−	+	−	−	−

Code family	Dental abnormalities			Laboratory findings			Imaging findings				Nerve conduction velocities	Other findings
	Caries	Tooth enamel abnormalities	Morphological tooth abnormalities	AST (NV < 40 U/l)	ALT (NV < 40 U/l)	CSF protein level (NV < 0.4gr/l)	Calcifications	Hypomyelination	Cerebellar atrophy	Brainstem atrophy		
*CS1*	*+*	*+*	*+*	964*	1780*	*0.75 g/l*	*+*	*−*	*+*	*−*	*NL (45 m/s)*	
*CS1*	*−*	*−*	*−*	*56*	*48*	*NA*	*+*	*−*	*−*	*−*	*NL (45 m/s)*	*Cryptorchidism*
CS2	−	−	−	62	91	NA	+	+	+	−	Slightly slowed 36 m/s	
*CS6*	*+*	*+*	*+*	*40*	*60*	*NA*	*+*	*−*	*−*	*−*	*NL (45 m/s)*	*Cryptorchidism*
*CS6*	*−*	*−*	*−*	*41*	*46*	*NA*	*+*	*NA*	*NA*	*NA*	*NA*	
CS7	+	−	−	41	47	NA	+	+	+	−	Slowed 23 −35 m/s	
CS11	−	−	−	37	32	NA	+	+	−	−	Slowed 13–32 m/s	
CS16	+	−	+	30	32	NA	NA	+	+	+	Slowed 17–31 m/s	

Patients from the same family are underscored in italics

*AST/ALT: tested twice (1 and 3 years old) the value normalized at the age of 3 years (47/53); *NA* not available, *NL* normal, *SD* standard deviation, *GD* growth delay, *PMD* psychomotor disturbance

#### General presentation of the patients

**Fig. 1:**
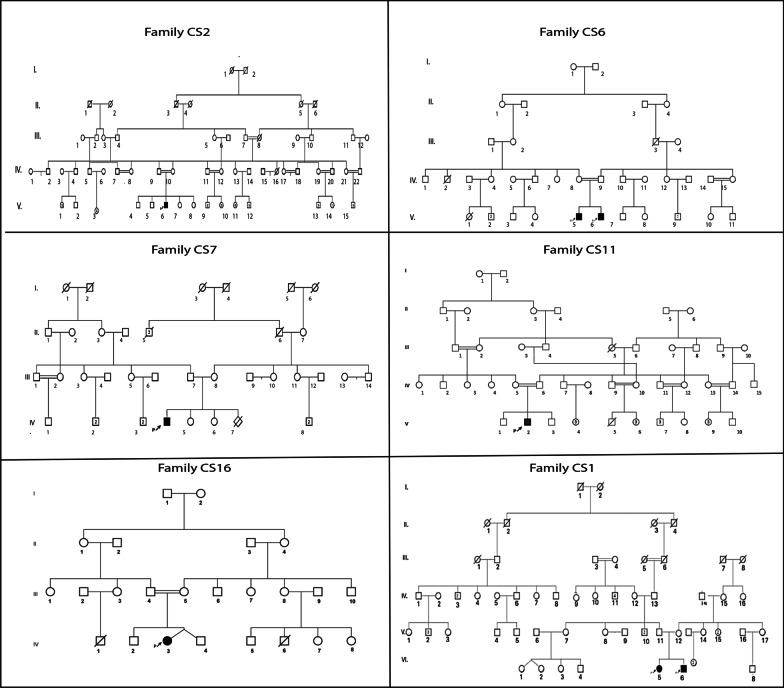
Pedigree of the six unrelated Tunisian families. The studied proband is indicated with an arrow

This cohort included 6 males and 2 females originated from six unrelated families (CS1, CS2, CS6, CS7, CS11, and CS16). Consanguinity, examined by genealogical data, was found in 4 families (CS2, CS6, CS11, and CS16), and endogamy was reported for two other families (CS1 and CS7). All patients originated from the North West of Tunisia except CS2 that originated from South Tunisia. The mean age of patients at the time of examination was 3.4 years ranging from 1.5 to 7 years. At the time of the study, all patients were alive (Fig. 1).

#### Post- and pre-natal abnormalities

Intra-uterine growth retardation (IUGR) was noted in four patients (CS1EA1, CS1EA2, CS11, CS16). No prenatal malformation was detected in ultrasound screening for seven out of the eight CS patients, except CS1EA2 who displayed microcephaly (Table 1). Delivery was at full term for all patients except CS16 who was born in the eighth month of pregnancy. No perinatal asphyxia was reported. Birth weight was within the low normal range for all patients (mean birth weight: 2575 g, ranging from 1450 g (as for CS16) to 3400 g). Based on the head circumference at birth, all patients except CS16 and CS1EA2 (n = 6) were normocephalic (mean birth head circumference: 33.28 cm). Microcephaly was reported for CS1EA2 in the clinical records but the value was not indicated. Postnatally, all patients developed progressive growth failure and microcephaly (mean head circumference -3SD: standard deviation defined according to growth curve) (Table 1), (Additional file 1: Figure S1).

#### Behavioral abnormalities, muscular neurological and neurosensory problems

All patients that arrived in our department for examination displayed psychomotor delay. Five patients were able to sit independently at a mean age of 13 months whereas two patients (CS11 and CS 16) were incapable to do so. Walking without support was acquired in two cases (CS6EA1et CS7) at 2 and 3 years, respectively, but the walking capacity was lost as the syndrome progressed (mainly due to contractures). Another patient (CS1EA1) was capable of walking with support at 30 months without reported abnormalities. The five other patients were unable to walk alone.

None of the patients had language skills at the time of examination. However, they were outgoing and interactive. Behavioral disturbance with irritability and sleep disorder were not reported except for patient CS1EA1. Neurological examination showed a spasticity in lower limbs in all patients leading to a progressive flexion contracture in six patients, associated with ataxia and tremor in three cases (CS1EA1, CS7, CS11), and neurological signs in one case (CS11, age 4). No patient had extrapyramidal signs. Kyphosis was noted in two patients (CS16 and CS1EA2). In three patients, the severity of contractures needed surgical intervention (CS1EA2, CS11, and CS16). Sensorineural deafness was detected in seven cases (all patients except CS11). Ophthalmological examination performed in five patients showed bilateral cataracts in four cases (CS1EA2, CS7, CS11, and CS16), and pigmentary retinopathy in one case (CS2).

#### Facial, dental, and skin anomalies

All patients had the characteristic facial appearance of CS with enophthalmia, large ears, and thin skin. Bird-like nose was noted in five patients (all patients except CS1EA2, and both CS6 patients). This facial phenotype was generally more evident in older patients (5–7 years old). Dental caries were observed in four cases (CS1EA1, CS6EA1, CS7, and CS16). Anomalies in tooth shape, size and number were reported in three patients (CS1EA1, CS6EA1, CS16). Photosensitivity was observed in five out of eight patients (not detected in the two CS1 siblings and the CS11 patient). Pigmentation abnormalities were observed in four patients (siblings of the CS1 and CS6 families).

#### Laboratory investigations

Mild serum aminotransferase elevation (> 2 N) was noted in all patients before the age of three, except CS11 and CS16. The biochemical analysis of aspartate aminotransferase/alanine aminotransferase (AST/ALT) was done longitudinally for the CS1EA1 patient, who showed cytolysis (964/1780) at the age of one, but her values normalized progressively at the age of three (47/53) (Table 1).

Cerebrospinal Fluid (CSF) analysis was performed in the CS1EA1 patient, who showed a mild increase of the CSF lactate level (2.13 mmol/l; normal value < 2). This patient also displayed a slight increase of creatinine kinase (824 UI/l; normal value < 145).

#### Neuroimaging analysis

**Fig. 2:**
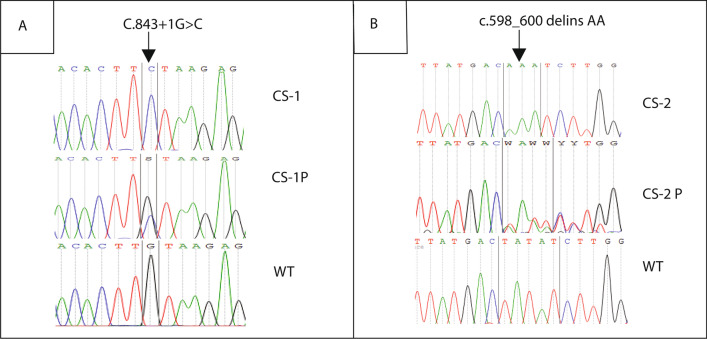
Genetic analysis of genomic DNA. Electropherogram showing: **A** the splice site mutation (c.843 + 1 G > C) in the *ERCC8* gene in CS-1 family (patient CS-1 and parent CS-1P) and **B** the mutation (c.598_600delinsAA) in the *ERCC8* gene in CS-2 family (patient CS-2 and parent CS-2P), compared to WT

Computed tomography was performed for seven patients (except CS16) and showed lenticular calcifications in all of them. Magnetic resonance imaging (MRI) was performed in these patients as well, showing hypomyelination in CS2, CS7, CS11, CS16, and cerebellar atrophy in CS1EA1, CS2, CS7, CS16 (Table 1). Cerebral MRI images of CS7 illustrate the white matter anomalies (Fig. 2).

#### Neurophysiological studies

Nerve conduction velocities were studied in seven patients, and values were low (< 45 m/s) in four of them (Table 1). Electroretinogram was performed once and was normal for all patients except CS2 (not shown).

### Genetic analysis

#### Genetic analysis reveals the same mutation in six patients

**Fig. 3:**
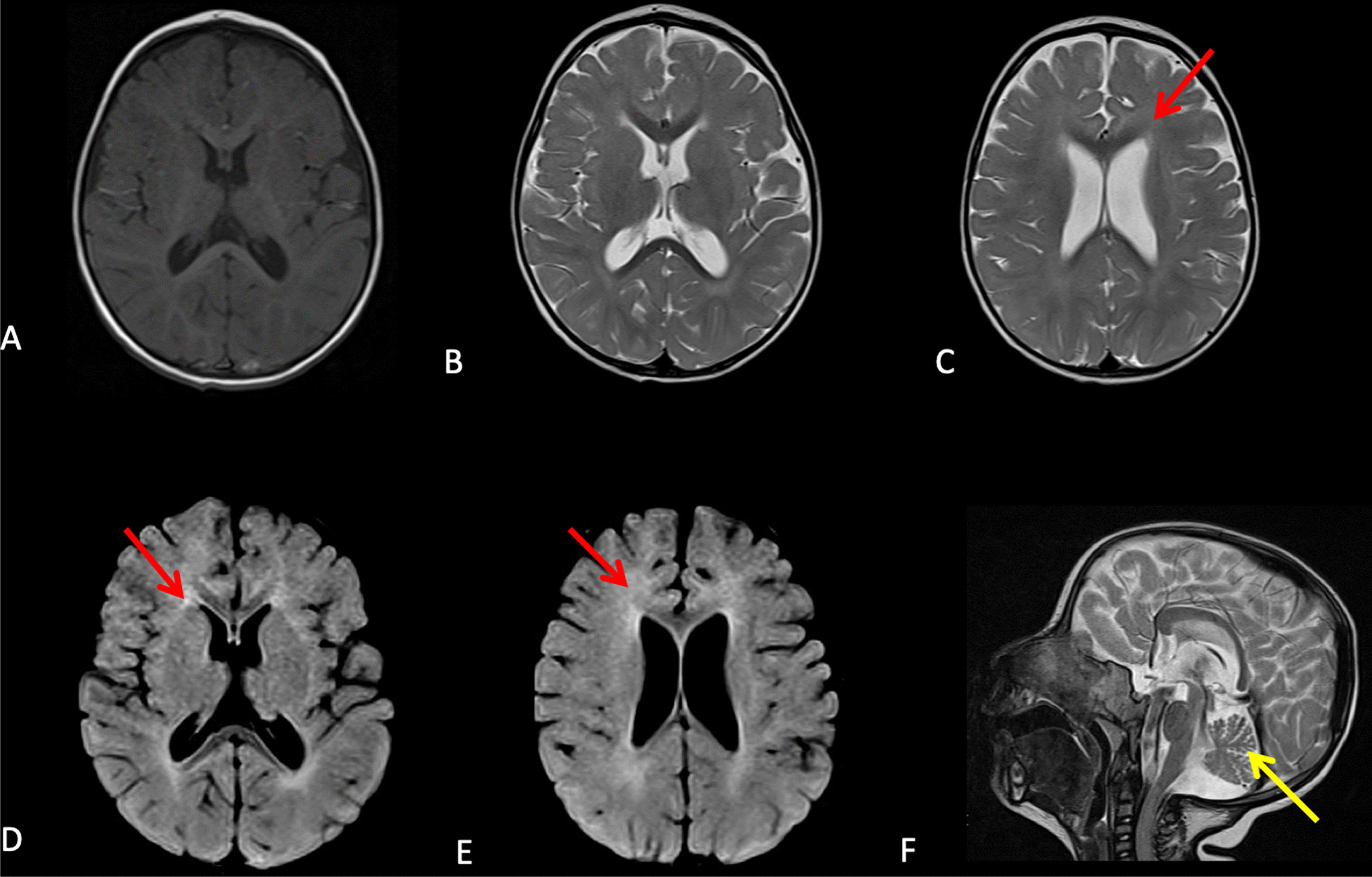
MRI image of CS7. **A** Axial T1 weighted-image, **B**, **C** axial T2 weighted-images, and **D**, **E** axial FLAIR weighted-images showing isointensity of periventricular white matter on T1, and hyperintensity on T2. FLAIR is suggestive of hypomyelinating leukodystrophy (red arrows). **F** Sagittal T2 weighted-image showing cerebellar atrophy (yellow arrow)

We first screened the eight patients with Sanger sequencing of *ERCC8* exon 7 (NM_000082.3), which revealed that six (CS2, both CS6, CS7, CS11, and CS16) out of eight CS patients were homozygous carriers for the variant c. 598_600delinsAA; p.(Tyr200Lysfs*12). This variant that introduces a stop codon and therefore a truncated protein has been previously described in several unrelated CS patients from North Africa [14, 15] (and unpublished data). We confirmed parental segregation of the mutation in all cases (Fig. 3). Conversely, this recurrent variant was absent in the affected siblings of the CS1 family.

#### Genetic analysis of the CS1 family

##### Identification of an intronic variant via targeted gene sequencing

In one patient of the CS1A family, screening of 17 genes involved in NER pathway using targeted gene sequencing revealed a homozygous transversion in *ERCC8* at the position of the locus reference genomic (LRG)_466t1: c.843 + 1G > C (Fig. 3). This variant represents a transversion from a guanine to a cytosine at the donor splice site of intron 9, and has been previously reported in a LebaneseCS patient [16]. Sanger sequencing confirmed that this mutation was homozygous in the two affected siblings in the CS1 family, and heterozygous in their parents, as expected.

##### In silico effect of the variant on splicing site

The variant modified the consensus donor splice site region in intron 9 of *ERCC8,* changing the conserved GT to a CT motif. This mutation is expected to alter the mRNA splicing by affecting the donor site signal, according to the prediction tools: Human Splicing Finder (HSF) and MaxEntScan. In particular, using HSF the potential impact of this variant was assessed through attribution of consensus value (CV) according to the matrices from Shapiro and Senepathy [17]. The difference between the wild type and the mutant had a CV of (− 32.71%), and was predicted by the program to abolish the donor site, thereby affecting the splicing process.

##### cDNA analysis of the splice site mutation

**Fig. 4:**
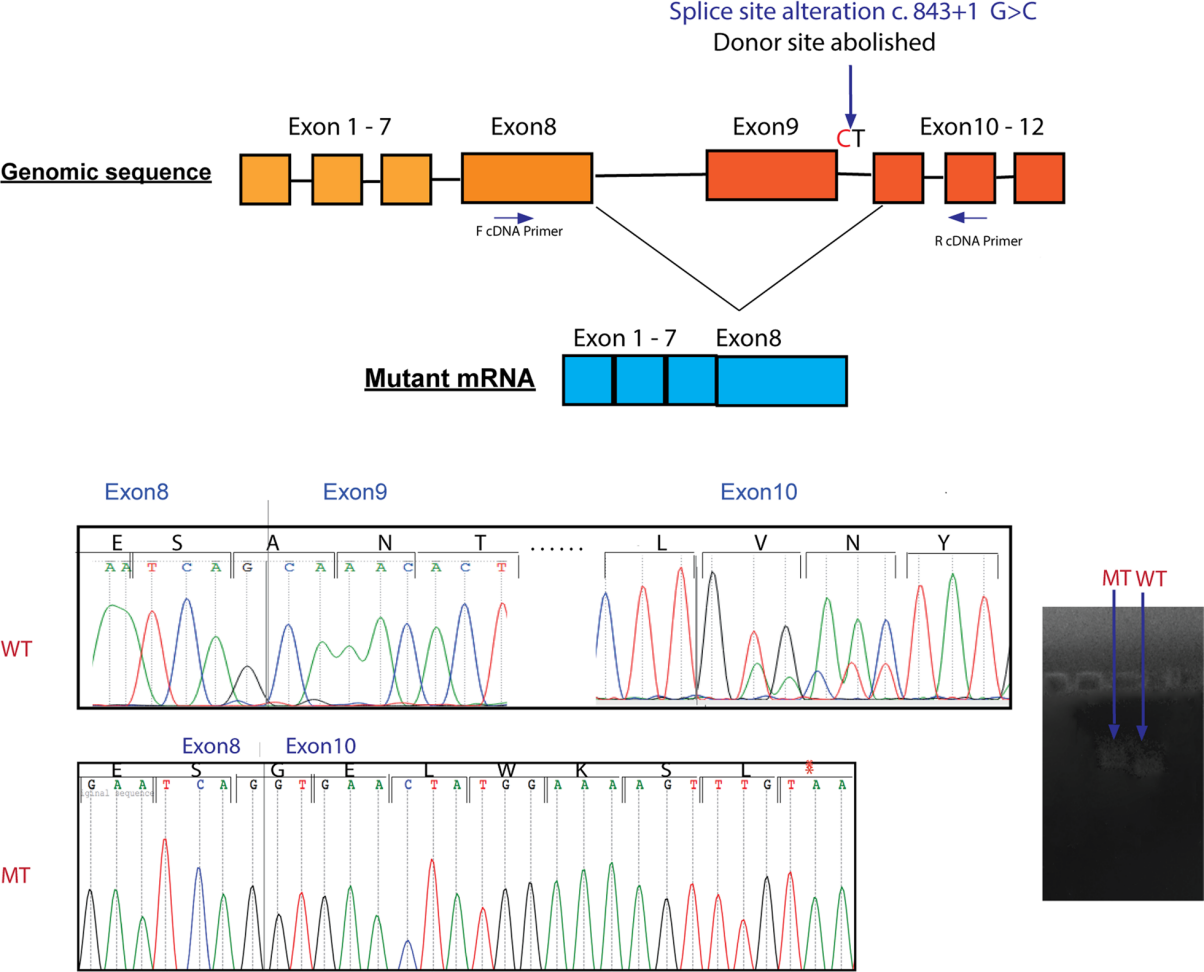
CSA splicing alterations. Upper panel, schematic representation of the *ERCC8* gene with aberrant skipping of exon 9 as a result of the c.843 + 1G > C mutation. Lower panel, sequencing of the mutant transcript (MT), which confirmed the aberrant splicing event compared to the wild type (WT); the stop codon in the mutant is indicated by an asterisk

To confirm and functionally validate the mutation at the splice site, mRNA extracted from primary fibroblasts derived from the CS1EA1 patient and a healthy control were compared. PCR amplification of cDNA from exon 8 to exon 11, using appropriate primers, resulted in a shorter fragment in CS1EA1 compared to control. Sequence analysis of the amplicon revealed that exon 9 was missing in the transcript of the CS1EA1 patient. At the protein level, skipping of exon 9 was predicted to shift the reading frame leading to the emergence of a premature stop codon eight amino acids downstream p.(Ala240Glyfs*8), and therefore resulting in a protein of only 246 aminoacids (aa) in length (instead of 396 aa) (Fig. 4).

### Cellular response to UV in CS patients

**Fig. 5:**
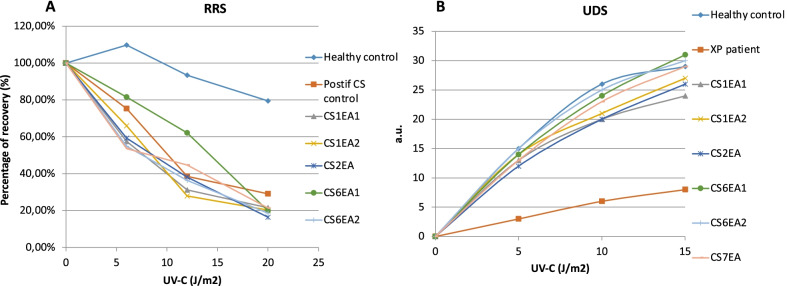
Response of UV radiation in fibroblasts from six CS Tunisian patients. **A** RRS 24 h after UV irradiation expressed in percentage of recovery after EdU incorporation showing the defect of RNA synthesis after UV exposure in CS fibroblasts. **B** Unscheduled DNA repair synthesis (UDS) expressed in arbitrary units (a.u.) of EdU fluorescence intensity. CS patients show a normal level of unscheduled DNA synthesis

UV irradiation tests performed on six cell lines derived from CS patients (CS1EA1, CS1EA2, CS2EA, CS6EA1, CS6EA2, CS7EA) showed reduced response to UV compared to healthy controls. Response to increasing doses (0–15 J/m^2^) of UV radiation was first assessed by RRS that displayed strongly reduced RNA synthesis in all tested CS samples compared to the healthy control, with a better response for CS6EA1 (Fig. 5). As expected, cells derived from CS patients displayed unscheduled DNA synthesis (UDS) comparable to values of healthy controls, whereas the XP positive control patient had low UDS levels (Fig. 5). Altogether these results indicate defective capacity to repair UV induced DNA damage on the transcribed strand in tested CS patients, including those that do not display abnormal sensitivity to sunlight (Table 1), in agreement with previous findings [4, 18].

## Discussion

### Mutations in eight CS patients

Mutation of essentially two genes has been associated with CS, namely *ERCC*6 in 68% and *ERCC*8 in 32% of patients [5]. The situation is possibly reversed in Tunisia and Arab countries, where *ERCC8* mutations seem to be more frequent [4, 15, 16, 19, 20]. The present study expands the clinical spectrum and increases the relevance of two *CSA* mutations. These genetic defects seem to be specific to the Tunisian and North African population, as they have not been reported elsewhere, at least to date. Indeed, since the first description of CS by Dr. Cockayne in 1936, only eleven patients have been reported in the Tunisian population: two siblings with one of the mutations described in the present study (c.598_600delinsAA) in *ERCC8/CSA* [14, 15], two other siblings with a private mutation (c.400-2A > G) in *ERCC8/CSA* [20], and three patients with the novel c3156dup mutation in *ERCC6/CSB* [10]. Four more CS patients have been clinically and biochemically characterized but their respective mutations have not been identified [21, 22].

In six patients of our cohort, Sanger sequencing identified a recurrent *ERCC8* variant, namely the homozygous mutation c.598_600delinsAA p.(Tyr200Lysfs*12), which was previously identified in two Tunisian siblings [14, 15]. *ERCC8* encodes a 44 kDa protein, CSA that contains 7 WD40 domains. Each of these domains is constituted by several WD [tryptophan (Trp, W), aspartic acid (Asp, D)] repeats. The c. 598_600delinsAA variant in *ERCC8* patients could lead to a nonsense-mediated mRNA decay (NMD). In detail, the alteration of the fourth evolutionarily conserved amino-acid residue in the WD4 repeated motif is predicted to result in a premature stop codon after 12 aminoacids. The WD motifs are required for the construction of the beta-propeller structure, which is important for protein complex formation and interactions of CSA with the transcription and repair factors DDB1, RNA polymerase II, TFIIH [13, 23].

The relatively larger proportion of *ERCC8* defects, and in particular the c.598_600delinsAA mutation, in Tunisian patients can be attributed to a founder effect. Further investigations including haplotype analysis are required to verify whether this is the case. Interestingly, one of the six patients had Algerian ancestries suggesting that this variant is a possible founder mutation in North Africa (Fig. 4).

Furthermore, via targeted gene sequencing, we detected in two patients (CS1EA1 and CS1EA2) a variant that has not been previously reported in the Tunisian population, i.e. c.843 + 1G > C*.* This homozygous mutation leads to the abolition of the consensus donor splice site in intron 9, generating a novel splice site, which leads to exon 9 skipping in the *ERCC*8 gene and the emergence of a premature stop codon. This donor splice mutation is predicted to generate a shorter protein lacking the last two WD40 domains, which may affect the function of this protein. This variant co-segregated in the CS1 family members, further supporting this variant as causal of the CS disorder in these patients.

The c.843 + 1G > C variant has been described in a CS patient from Lebanon [16], but the conclusions on the consequence of this variant on the transcript differ in our study. Indeed, Chelby et al. suggested that intron 9 (located between exons 9 and 10) was present in this variant because a PCR test with primers located in these two exons failed to amplify a fragment, indicating the presence of a long intron 9. However, one of the primers used in this PCR was located exactly in exon 9. In this case, the reason for lack of amplification was rather the absence of exon 9, in agreement with our findings. Moreover, the presence of intron 9 was not further demonstrated. Another possibility is that this transcript was not detected in the previous study because it is poorly expressed. In the absence of exon 9, the amplification obtained by Chelby et al. with a pair of primers englobing the region comprised between exon 9 to intron 9 could be due to contaminating DNA acting as a competitor in the PCR reaction [24], if samples were not treated with DNase before RT-PCR, as we did. According to our data, which are compatible with a splicing variant, this mutation has ultimately the same consequences as the c.843 + 2T > G and c.843 + 5G > C variants that have also been suggested to alter donor splice site and lead to a premature stop codon p.(Ala240Glyfs*8) [14, 25].

### Remarkable clinical features and lack of clinical photosensitivity

Each of the reported cases in the present study displays distinct clinical features. It is worth to note that some patients (CS1 siblings, CS11, and CS16) suffered from intra-uterine growth retardation. This clinical feature is more frequently associated with the severe form of CS type II, which is usually linked to mutation in *ERCC6.* Conversely, all patients of this study were linked to the *ERCC*8 gene, which is normally associated with less severe forms [18, 26]. Other clinical manifestations as microcephaly and ataxia at birth are not specific to CS, and have been also described in mitochondria-associated diseases, which makes the CS diagnosis more difficult at early stages.

Previous studies reported CS patients that do not present clinical photosensitivity, as in Tunisian, Turkish, Italian, and Moroccan populations [4, 21, 27, 28]. Therefore, cutaneous photosensitivity was classified as a minor criterion in the diagnosis of CS as it appears in about 75% of patients, and was not correlated with the type of genetic defect in the TCR-NER pathway. Our data, with two siblings from the CS1 family (mutation c.843 + 1G > C), as well as the CS11 patient (mutation c.598_600delinsAA) not displaying clinical photosensitivity confirm that this defect is not an essential criterium for CS. The absence of clinical photosensitivity required to assess whether the repair of UV-induced DNA damage by TC-NER in primary fibroblasts from these patients was affected. Indeed, fibroblasts from CS patients have increased sensitivity to UV irradiation [29], indipendently of the extent of clincal photosensitivity. Conventional methods to assess TC-NER include RRS following UV damage that is impaired in CS [30], and UDS that is not affected in these patients whereas it is in XP patients [31]. To be noticed, when clinical photosensitivity is identified in CS, it remains rather moderate compared to other forms of genodermatosis related to defects of the NER system.

In the present study, conventional mild phenotype CS patients as well as CS patients who did not show photosensitivity displayed similarly low RRS values compared to healthy controls. This result confirms that photosensitivity, although not clinically visible, is present at the cellular level in these patients.

Altogether these findings further substantiate that Cockayne syndrome may not be solely accounted for the defective NER system. Indeed, variants in *ERCC6* and *ERCC8* genes have been also associated with the UV sensitive syndrome (UVSS), a milder form clinically characterized by mild cutaneous symptoms [32]. In UVSS patients, reduced RRS after UV radiations was also observed, indicating that the TC-NER impairment did not lead to neurodegeneration or premature ageing as it is the case in CS.

Lack of association between CS and clinical photosensitivity in some patients suggests that other or additional mechanisms than the DNA repair defect are involved in the etiology of CS. In this context, CS exhibit altered mitochondrial metabolism and an accumulation of oxidative stress at the cellular level [33, 34]. CSA and CSB are indeed multifunctional proteins that are involved in several processes in addition to DNA repair [35, 36].

### Heterogeneous clinical features in patients with the same mutation and siblings

CS is a clinically heterogeneous disease and is caused by a large number of distinct mutations in *ERCC6* or *ERCC8* [4, 9]. For comparison, other monogenic diseases, for instance the Hutchinson-Guilford progeria syndrome (HGPS) is mostly due to a single point mutation that activates an alternative splicing site that produces an altered form of the lamin A protein [37]. Conversely, 38 pathogenic variants have been described just for *ERCC8/CSA* and which concern totally 84 CS patients [9]. Since genotype/phenotype correlation remains elusive in CS, relevant information may originate from the assessment of clinical symptoms in multiple patients and, when available, siblings carrying the same mutation. However, this situation is rather infrequent, and only three other cases of siblings [15, 20, 38], and a few cases of patients carrying the same mutations [10] have been described in CS. The present study that reports a detailed clinical characterization of six patients, including two siblings that carry the same mutation, as well as two other siblings carrying another mutation, represents a powerful data set to address this question.

The six patients carrying the c.598_600delinsAA mutation shared common characteristics: early age symptoms [0–24 months], prenatal abnormalities as microcephaly, cerebellar hypoplasia, olighydramnios, and lower post-natal weight and height. They also displayed different combinations (presence/absence) of other defects like normal or low birth weight and height, ataxia, cataracts, dental abnormalities, hypomyelination, cerebellar atrophy, etc. Importantly, within this group the two CS6 siblings displayed remarkable phenotypic differences concerning for instance post-natal height, independent walking, dental abnormalities, and cryptorchidism.

The two siblings from the CS1 family (mutation c.843 + 1G > C) presented high levels of transaminase which are commonly observed in other CS patients, possibly reflecting a mild liver damage [3, 39]. Moreover, the younger of the two patients displayed severe symptoms like the emergence of cataracts at an early age. Indeed, the presence of cataracts is normally associated with a worst probability of survival, and death before the age of 7 for CS patients [40]. Only one of the two siblings (CS1EA1, a male) showed prenatal microcephaly, olighydramnios, and cataracts. Conversely, only the other sibling (CS1EA2, a female) showed bird-like nose dysmorphism, limb spasticity, ataxia, hair and dental abnormalities, cerebellar atrophy. These clinical differences in the context of the same mutation and, in the case of siblings also of comparable genetic backgrounds, underscore the large heterogeneity of CS clinical symptoms that is difficult to reconcile with a simple genotype/phenotype alteration, and the reason of which remains obscure.

It is important to note that the clinical heterogeneity of patients that share the same recurrent mutation increases the difficulty for clinicians to confirm the clinical diagnosis of this disease, and may generate confusion with pathologies that display related symptoms like those linked to mitochondrial etiopathology such as mitochondrial cytopathies. Moreover, the clinical heterogeneity in CS may represent a further challenge for treatments, which have not been developed for CS to date.

### Characteristics of the CS-A cohort

We reported six patients with the same homozygous variant, including one that appeared to have an Algerian ancestry (according to the genealogical questionnaire). This mutation was previously observed in two other Tunisian patients [41], which suggests that it is a founder mutation in the region. The CS6 siblings were born from a consanguineous marriage. Although the CS1 siblings were born from a non-consanguineous marriage, the emergence of the homozygous mutation, and thereby of CS, is likely due to the high rate of endogamy in this region. In Tunisia, the high rate of endogamy contributes to the increased risk (96.64%) of recessive diseases in isolated communities even without consanguinity [42].

The two siblings of the CS1 family harbor the same genetic variant as in a previously reported Lebanese patient, who also displayed a severe CS phenotype [16]. North Africa's abundant prehistoric and historic cultural heritage has contributed to the diversity of the genetic pool of its population nowadays [43]. This pool originates from a combination of Middle Eastern, sub- Saharan Africa and Western European genetic components. For instance, the two Tunisian CS1 patients described here share a variant with the Lebanese patient born from Druze parents, possibly dating back to a common ancestry. In fact, Druze was first reported under the Fatimid Dynasty, a dynasty that originated in Tunisia and spread to some region of Middle East [44]. Druze is a closed community with high rate of inbreeding (around 53%), which has increased the rate of autosomal recessive diseases [45].

## Conclusions

In the present work we report the largest cohort of patients with Cockayne syndrome due to *ERCC8/CSA* mutations in Tunisia and North Africa, and enlarged the description of *ERCC8/CSA* variants globally. This study provides genetic, biochemical, and clinical data on siblings and multiple patients carrying the same *ERCC8/CSA* variant, underscoring the large heterogeneity of CS beyond the mutation. Although all CS-derived cells explored in this work had a DNA repair defect following UV exposure, some patients including those with a severe phenotype, did not show clinical photosensitivity. This finding confirms the notion that photosensitivity is not an essential clinical feature of this pathology, and further questions the mechanistic link between some clinical manifestations and the deficit of the DNA repair system.

A thorough clinical characterization in CS patients, in whom the deleterious effect of the identified mutations has been confirmed, should facilitate the early follow-up of other patients and the establishment of a prenatal diagnosis. Indeed, thanks to the collaboration between clinicians and researchers in the frame of our study, three prenatal diagnosis were carried out for two consanguineous families at risk (the CS1 and CS6 families).

## Methods

### Patients

Eight patients were recruited from the Department of Child Neurology (National Institute Mongi Ben Hmida de Tunis) in 2017–2019. These patients underwent neurological and general examination routine since 2017. Blood tests, metabolic tests, CT-scan and/or brain MRI and electrophysiological studies have been done for patients strongly suspected to be affected by Cockayne syndrome. Written informed consent was obtained from patients’ parents as CS patients were minors. Blood and skin biopsies as well as genealogical data were collected. The study was approved by the Institut Pasteur de Tunis (IPT) Biomedical Ethics Committee in Tunisia (reference 2017/31/I/LR16IPT05/V2), in accordance with the Declaration of Helsinki Principles.

### DNA extraction and quantification

Genomic DNAs were isolated from peripheral blood of patients and their parents using FlexiGene kit (Qiagen). DNA samples yield and purity were assessed using a Nanodrop Spectrophotometer (Thermo Scientific, Wilmington, USA).

### gDNA sequencing

Genetic studies started by screening for the recurrent *ERCC8* pathogenic variant already described in North African CS patients NM_000082.3: c.598_600delinsAA; p.(Tyr200Lysfs*12) using Sanger sequencing (F: 5′ CAAGTGATGGACTTCACCTC 3′; R: 5′ CTGCCTGAACATCCCTAATC 3′). *ERCC*8 exon 7 was amplified with the following primers set (F: 5′ CCCTTTGAACTTATCACCTG 3′; R: 5′ CCTCTGTGTCCCTAGCACAAT 3’) and sequenced using the ABI 3130 Genetic Analyzer (Applied Biosystems).

In absence of the recurrent variant, molecular screening of the patients was continued by NGS assay targeting 17 genes involved in the NER pathway (*DDB2, ERCC1, ERCC2, ERCC3, ERCC4, ERCC5, ERCC6, ERCC8, GTF2H5, MPLKIP, PCNA, POLH, RNF113A, SMARCAL1, UVSSA, XPA, and XPC*). Regions of interest were captured using SureSelect QXT Agilent probes and libraries were sequenced on a NextSeq550 Illumina platform [46].

For data analysis, home-made “STARK” and Polyweb (Université Paris Descartes) pipelines were used to detect both single nucleotide and copy number variant [46]. We filtered and selected the variants whose minor allele frequency was inferior to 0.05 in dbSNP, HapMap, and 1000 Genome Project. Variants were subsequently characterized according to the American College of Medical Genetics and Genomics (ACMG) [47] and the filtering strategy of valuable variants was similar to protocols reported in previous studies [46, 48, 49]. Pathogenicity of the variants were tested using online prediction tools like MutationTaster (http://www.mutationtaster.org/), Sift (https://sift.bii.a-star.edu.sg/), Polyphen (http://genetics.bwh.harvard.edu/pph2/), MaxEnScan (http://hollywood.mit.edu/burgelab/maxent/Xmaxentscan_scoreseq_acc.html) and previously publically available Human Splicing Finder version 3.1_2017 (http://www.umd.be/HSF/). The presence of a variant in a proband was confirmed by Sanger sequencing as well as segregation analysis.

### Primary dermal fibroblasts

All dermal fibroblasts were obtained from skin biopsies. Cells were grown at 37 °C in 5% CO_2_ humidified atmosphere in Dulbecco’s modified Eagle medium (DMEM) (1 g/L glucose) w/GLUTAMAX (Life Technologies, Gibco) supplemented with 10% of fetal calf serum (Gibco) and 1% penicillin/streptomycin (Gibco). All fibroblasts primary cultures were assessed at comparable passage number (passage number 3–4).

### Analysis of *ERCC*8 cDNA from primary dermal fibroblasts cultures

Total RNA from 10^6^ of dermal fibroblasts was isolated using Trizol reagent (Sigma-Aldrich) according to the manufacture’s instruction. To avoid contamination with genomic DNA, samples were treated with DNase (invitrogen). The cDNA was synthesized from 1 µg of RNA using oligo dT primers with the Superscript Reverse transcriptase II (Invitrogen), according to the manufacturer’s instructions. For the analysis of the region of interest, polymerase chain reaction was used to amplify the cDNA spanning exon 8 to exon 12 (F:5′ GTGAGAAGAGCATCAGGATG3′; R:5′ CCAGAATGTTGCAGTCTCTG3’). Which was assessed in agarose gel and compared to healthy for amplicon's length and further analyzed via Sanger sequencing.

### DNA repair essay

Responses to UV irradiation in primary fibroblasts were evaluated through UDS and RRS analyses after DNA damage, as described [50–52]. Briefly, cells were plated on coverslips in 6-well plates and exposed to UV-C doses at 0, 5, 10 and 15 J/m^2^. De novo DNA synthesis was measured via incorporation of 5-ethynyl-2'-deoxyuridine (EdU) after UV irradiation in 6 CS patient fibroblasts (CS1EA1, CS1EA2, CS2, CS6EA1, CS6EA2, CS7), one healthy donor control, one Xeroderma pigmentosum and one Cockayne syndrome (affected DNA repair) controls. RNA detection was performed by irradiating primary culture of fibroblasts with UV-C doses (0, 6, 12, and 20 J/m2). 5-ethynyl uridine (5-EU) incorporation was assessed after 24 h of recovery from the UV exposure. The images were processed and analyzed with Image J for 50 randomly selected cells, originating from three independent experiments, and the average nuclear fluorescence intensity was calculated.

## Supplementary Information

**Additional file 1**. Growth chart for CSA male patients (0-5 years) compared to the WHO reference charts (mean, 3rd and 97th percentile). (A) Weight, (B) height, and (C) occipital frontal circumference.

## Data Availability

All processed data have been provided in the manuscript. Raw data, generated for this study could be provided by the corresponding author upon reasonable request.
