# Supplementary figures and images for: Heterogeneous clinical features in Cockayne syndrome patients and siblings carrying the same CSA mutations
Source: Orphanet J Rare Dis. 2022 Mar 5;17:121. doi: 10.1186/s13023-022-02257-1 (PMC8898519; doi:10.1186/s13023-022-02257-1)

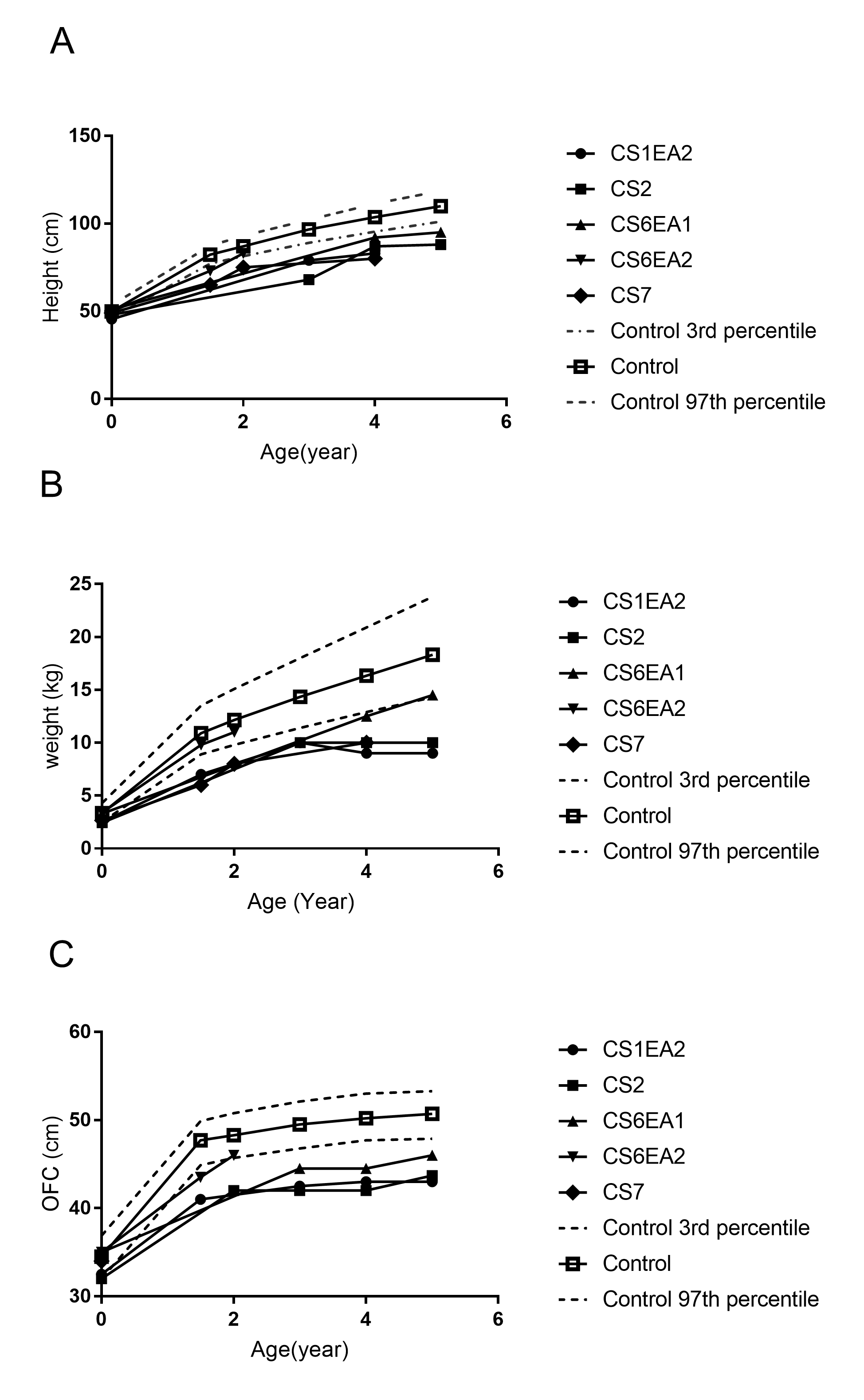

Supplement: Supplementary file 1 — Additional file 1. Growth chart for CSA male patients (0-5 years) compared to the WHO reference charts (mean, 3rd and 97th percentile). (A) Weight, (B) height, and (C) occipital frontal circumference. [file 13023_2022_2257_MOESM1_ESM.tif]
